# High Plasma sTNF-R1 Level Is Related to Loss of Natural HIV Control in Long-Term Elite Controllers

**DOI:** 10.3389/fcimb.2022.858872

**Published:** 2022-03-17

**Authors:** Daniel Sepúlveda-Crespo, Norma Rallón, María José Muñoz-Gómez, Oscar Brochado-Kith, José Luis Jiménez, María Ángeles Muñoz-Fernández, José M. Benito, Salvador Resino

**Affiliations:** ^1^ Unidad de Infección Viral e Inmunidad, Centro Nacional de Microbiología, Instituto de Salud Carlos III, Majadahonda, Spain; ^2^ HIV and Viral Hepatitis Research Laboratory, Instituto de Investigación Sanitaria Fundación Jiménez Díaz, Universidad Autónoma de Madrid (IIS-FJD, UAM), Madrid, Spain; ^3^ Hospital Universitario Rey Juan Carlos, Móstoles, Spain; ^4^ Plataforma de Laboratorio, Hospital General Universitario Gregorio Marañón (HGUGM), Madrid, Spain; ^5^ Spanish HIV HGM Biobank, Madrid, Spain; ^6^ Instituto de Investigación Sanitaria del Gregorio Marañón (IiSGM), Madrid, Spain; ^7^ Sección Inmunología, Laboratorio InmunoBiología Molecular, Hospital General Universitario Gregorio Marañón (HGUGM), Madrid, Spain

**Keywords:** HIV-1, long-term elite controllers, plasma biomarkers, inflammation, AIDS progression

## Abstract

Human immunodeficiency virus-1 (HIV-1) elite controllers are heterogeneous due to different immunovirological features. We aimed to identify plasma biomarkers associated with loss of spontaneous HIV-1 control in long-term elite controllers (HIV-LTECs). We performed a retrospective study in 60 HIV-LTECs [36 true-LTECs and 24 LTECs losing control (LTECs-LC)]. We selected a plasma sample from true-LTECs (towards the middle of the follow-up period) and two samples from LTECs-LC (one far from the loss of control and another close to loss of control). Plasma biomarkers were evaluated using multiplex immunoassays. The partial least squares-discriminant analysis provided the variable importance in projection (VIP), and the adjusted Generalized Linear Model provided the adjusted arithmetic mean ratio (aAMR). At the moment of the first LTECs-LC samples, the only plasma biomarker with a VIP≥1.5 was sTNF-R1, which showed higher values in LTECs-LC than true-LTECs [aAMR=1.62 (95%CI=1.20-2.19); p=0.001]. After a median of 3.9 (IQR=4.5) years of follow-up from the first sample, we also had access to a second plasma sample from 10 LTECs-LC patients. At the moment of this second LTECs-LC sample, the only plasma biomarker with VIP≥1.5 was also sTNF-R1, which showed higher values in LTECs-LC than true-LTECs [aAMR=1.93 (95%CI=1.41-2.65); p<0.001]. The difference between the first and second samples of LTECs-LC was significant (Δx= 6.58 (95%=0.3; 12.88); p=0.040). In conclusion, high plasma values of sTNF-R1 appear to discriminate HIV-LTECs that lose the natural control of HIV-1, helping to define a specific phenotype that may be useful for the clinical management of these patients.

## Introduction

Human immunodeficiency virus-1 (HIV-1) infection promotes an immune dysfunction characterized by an exacerbated systemic immune activation and chronic inflammation leading to the loss of CD4^+^ T-cells and the progression to acquired immunodeficiency syndrome (AIDS) (Okoye and Picker, 2013). However, a minority group (<1%) of people infected with HIV-1 can spontaneously control HIV-1 infection and delay progression to AIDS (Navarrete-Munoz et al., 2020). They are named HIV-1 elite controllers (HIV-ECs) (Gebara et al., 2019). According to the International HIV Controller Consortium, HIV-ECs are patients maintaining undetectable plasma viral loads (<50-75 HIV-1 RNA copies/mL) for at least one year in the absence of antiretroviral therapy (ART) (Deeks and Walker, 2007). However, HIV-ECs are heterogeneous due to different virological, immunological, and clinical features (Navarrete-Munoz et al., 2020). Long-term elite controllers (HIV-LTECs) have recently been defined as a model to find the spontaneous functional HIV-1 cure within this heterogeneous group of patients (Navarrete-Munoz et al., 2020; Casado et al., 2020). HIV-LTECs is an extreme phenotype with a sustained virological and immunological control in the long term (Navarrete-Munoz et al., 2020). However, HIV-ECs and HIV-LTECs may lose virological and immunological control, leading to immunological dysfunction and AIDS progression.

There are specific factors linked to eventual loss of HIV-1 control (Deeks and Walker, 2007; Navarrete-Munoz et al., 2020; Ruiz-Mateos et al., 2020), including elevated activation and inflammation, characteristics of HIV-1 (diversity, envelope glycoprotein, proviral HIV-DNA, tropism, and superinfection), weak HIV-specific T cell responses, among others. High levels of proinflammatory biomarkers, such as GROα, CCL11, RANTES, IP10, sTNF-R1, and IFN-α, have been related to losing HIV-1 control in HIV-ECs. However, these factors remain unclear, and there is very little information on this topic in the exceptional group of HIV-LTECs. The current study aimed to identify plasma biomarkers associated with loss of spontaneous HIV-1 control in HIV-LTECs.

## Material and Methods

### Patients

We performed a multicentre retrospective study in HIV-ECs without ART from the Spanish AIDS Research Network cohort of HIV-ECs (ECRIS cohort). The ECRIS cohort incorporates patients from the long-term nonprogressor (LTNPs) cohort, CoRIS cohort, and different hospitals (see **Appendix 1**) and has been already described (Leon et al., 2016; Dominguez-Molina et al., 2020).

The ECRIS cohort includes HIV-1 patients with at least three consecutive data of plasma HIV-1 viral load (pVL) under the detection limit (pVL<50 copies/mL) during at least 12 months of follow-up. From this ECRIS cohort, we selected all HIV-ECs with long-term control of HIV-1 replication (defined as a minimum of 5 years), forming the group of HIV-LTECs that was divided into two groups according to the immunological and virological control during the whole period of follow-up available in the ECRIS database: a) 36 HIV-LTECs maintaining immunological and virological control throughout the whole follow-up (termed as true-LTECs); b) 24 HIV-LTECs that lost virological and/or immunological control during follow-up (termed LTECs losing control; LTECs-LC). In this study, the loss of virological control was defined as two consecutive measurements of pVL above the lower detection limit. Therefore, the existence of viral blips was not an exclusion criterion for true-LTECs, as explained in Leon et al. (2016) for the ECRIS cohort. The loss of immunological control was defined as a statistically significant negative slope of CD4^+^ T-cells count during the follow-up period.

The study was conducted following the Declaration of Helsinki, and all participants signed written consent. Besides, the Institutional Review Boards of the participating hospitals and Spanish HIV HGM BioBank approved this study. This study was approved by the “Fundación Jiménez Díaz” Ethics Committee (Ref.: PIC097-19_FJD).

### Samples

Peripheral venous blood samples were collected using ethylenediaminetetraacetic acid (EDTA) tubes, which were sent on the day to the Spanish HIV HGM BioBank (http://hivhgmbiobank.com/?lang=en), and immediately processed and stored at -80°C (Garcia-Merino et al., 2009).

We selected a plasma sample from true-LTECs patients (towards the middle of the follow-up period) and two samples from LTECs-LC patients when the patient had not yet lost spontaneous HIV-1 control, one far and one close to loss of control, except in 3 patients whose plasma sample coincided with a blip because no other plasma sample was available. The Spanish HIV HGM BioBank kindly provided patients samples.

### Multiplex ELISA

Plasma biomarkers [GROα (*CXCL1*), IL-8 (*CXCL8*), MIG (*CXCL9*), IP-10 (*CXCL10*), ITAC (*CXCL11*), SDF-1α (*CXCL12*), MCP-1 (*CCL2*), MIP-1α (*CCL3*), MIP-1β (*CCL4*), RANTES (*CCL5*), eotaxin (*CCL11*), sCD14, IL-1α, IL-1β, IL-18, IL-1RA, IL-6, sTNF-R1, sTNF-R2, and MMP9] were evaluated using ProcartaPlex Multiplex Immunoassay (Thermo Fisher Scientific Inc., Waltham, MA, USA) in a Bio-plex 200 system (BioRad Laboratories, Hercules, CA, USA). Due to the high proportion of samples being below the limit of detection, the values of crude fluorescence intensity (a.u., arbitrary units) were used as a relative quantification of the analyte concentration, as described previously (Breen et al., 2016).

### Statistical Analysis

The statistical analysis was performed with SPSS 25 (IBM Corp, Armonk, NY, USA) and R statistical package version v3.4.1 (R Foundation for Statistical Computing, Vienna, Austria). Figures were created using GraphPad Prism v8.0 (GraphPad Software, Inc., San Diego, CA, USA). All p-values were two-tailed and statistical significance was *p*<0.05.

Categorical data were analyzed by the chi-squared test or Fisher´s exact test, and continuous variable by Mann-Whitney U test for descriptive analysis. Moreover, we performed partial least squares-discriminant analysis (PLS-DA) [R-packages “mixomics v6.3.2”], a supervised multivariate analysis capable of dealing with multicollinearity for all plasma biomarkers. PLS-DA classifies biomarkers according to their variable importance in projection (VIP) score, which indicates the relevance of the biomarker in the discriminating model generated to sort each patient in the correct group (true-LTECs *vs.* LTECs-LC). Plasma biomarkers with VIP≥1.5 were selected as the most relevant for further analysis. Next, we used Generalized Linear Models (GLM) and GLM mixed with a gamma distribution (log-link) to evaluate the differences in plasma biomarker levels. GLM models were adjusted by patient characteristics (age, gender, hepatitis C virus (HCV) coinfection, hepatitis B virus (HBV) coinfection, HIV-1 transmission, time as EC, nadir CD4^+^, and HIV-RNA blips), which were selected by the stepwise algorithm (*p*-value <0.20) to avoid the over-fitting of the regression. This test gives us the adjusted arithmetic mean ratio (aAMR), 95% confidence interval (95%CI), and p-values.

## Results

### Study Population

The characteristics of the 60 HIV-LTECs (36 true-LTECs and 24 LTECs-LC) are shown in **Table 1**. Overall, the median age was 46 years, 55% were male, more than 70% were HCV or HBV coinfected, and 60% were injecting drug users. Nadir CD4^+^ was 491 cells/µL, but true-LTECs had higher values than LTECs-LC (*p*=0.018).

**Table 1:** Characteristics of HIV-infected patients included in the study.

Characteristic	All patients	True-LTECs	LTECs-LC	p-value
**No.**	60	36	24	
**Age (years)**	46.2 (39.8; 49.7)	45.4 (39.4; 49.2)	46.3 (42.3; 50.1)	0.415
**Gender (males)**	33 (55%)	22 (61.1%)	11 (45.8%)	0.244
**Country**				
** Spain**	51 (85%)	29 (80.6%)	22 (91.7%)	0.423
** Latin American**	4 (6.7%)	3 (8.3%)	1 (4.2%)	0.944
** Others**	5 (8.3%)	4 (11.1%)	1 (4.2%)	0.655
**Coinfections (%)**				
** Hepatitis C antibodies +**	43 (71.7%)	27 (75%)	16 (66.7%)	0.483
** Hepatitis C RNA +**	34 (56.7%)	21 (58.3%)	13 (54.2%)	0.750
** Hepatitis B antibodies +**	42 (70%)	26 (72.2%)	16 (66.7%)	0.826
** Hepatitis B DNA +**	5 (8.3%)	2 (5.6%)	3 (12.5%)	0.380
**HIV transmission route (%)**				
** IDU**	36 (60%)	23 (63.9%)	13 (54.2%)	0.451
** Homosexual transmission**	6 (10%)	4 (11.1%)	2 (8.3%)	0.999
** Heterosexual transmission**	14 (23.3%)	7 (19.4%)	7 (29.2%)	0.381
** Others**	4 (6.7%)	2 (5.6%)	2 (8.3%)	0.999
**Nadir CD4^+^ (cells/µl)**	491 (363; 676)	537 (439; 693)	389 (313; 540)	**0.018**
**Follow-up as HIV-ECs**				
** Time of follow-up (years)**	12.8 (7; 15.6)	12.6 (6.8; 15.2)	13 (8.6; 17)	0.271
** CD4^+^ count (cells/µl) at the beginning of follow-up**	873 (645; 1061)	784 (630; 1019)	916 (696; 1129)	0.212
** CD4^+^ count (cells/µl) at the end of follow-up**	783 (590; 1069)	878 (663; 1131)	650 (490; 827)	**0.017**
** HIV-RNA blips (pVL>50 cp/ml)**	32 (53.3%)	17 (47.2%)	15 (62.5%)	0.245
** % HIV-RNA blips (pVL>50 cp/ml)**	3.7 (0; 11.3)	0 (0; 8.1)	8 (0; 21.8)	0.093
**The first sample (n=60)**				
** HIV-RNA load (cp/ml)**	40.5 (24.5; 50)	40 (20; 50)	49.5 (37; 50)	0.372
** CD4^+^ count (cells/µl)**	861 (670; 1079)	879 (696.5; 1108)	797 (656; 1064)	0.350
**The second sample (n=10)**				
** HIV-RNA load (cp/ml)**			48.5 (28.5; 52)	–
** CD4^+^ count (cells/µl)**			602 (465; 775)	–
**Type of HIV loss of control**				
** Virological**	–	–	6 (25%)	–
** Immunological**	–	–	14 (58.3%)	–
** Virological and immunological**	–	–	4 (16.7%)	–

Statistic: Values for all participants and subgroups were expressed as median (percentile 25; percentile 75) for quantitative variables and absolute number (percentage) for qualitative variables. The statistically significant differences are shown in bold.

HIV, Human immunodeficiency virus; HCV, Hepatitis C virus; HBV, Hepatitis B virus; EC, elite controller; LTEC, long-term elite controller; LTEC-LC, LTEC losing control; pVL, plasma viral load; cp, copies; IDU, injected drug user.

During the follow-up as HIV-ECs (12.8 years), the median of the first CD4^+^ count was 873 cells/µL, and the last CD4^+^ count was 783 cells/µL. CD4^+^ count at the beginning of follow-up was similar in both groups of patients, whereas CD4^+^ count at the end of follow-up was significantly lower in LTECs-LC than true-LTECs (*p*=0.017).

### Plasma Biomarker in HIV-LTECs

**Figure 1:**
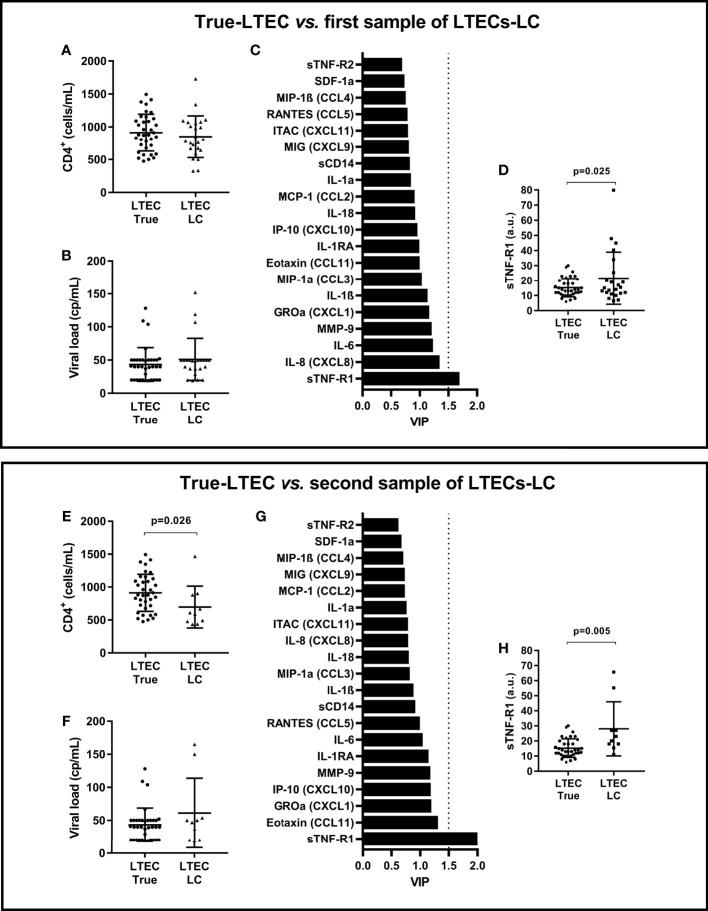
Summary of analysis between samples of 36 true-LTECs *vs.* 24 LTECs-LC. **(A, E)** CD4+ T-cell counts. **(B, F)** HIV-1 viral load levels. **(C, G)** Variable importance in projection (VIP) score from all analyzed plasma biomarkers. **(D, H)** Differences in sTNF-R1 between groups. LTECs, long-term elite controllers; true-LTECs, LTEC who did not lose virological and immunological control during follow-up; LTECs-LC, LTEC who lost virological and/or immunological control during follow-up; VIP, variable importance in projection; a.u., arbitrary units.

The type of HIV loss of control in LTECs-LC was 25% virological, 58.3% immunological, and 16.7% virological and immunological. At the moment of the first LTECs-LC sample, CD4^+^ T-cell count and HIV-1 viral load levels were similar between true-LTECs (n=36) and LTECs-LC (n=24) (**Figures 1A, B**). Using a PLS-DA, we found that sTNF-R1 was the only plasma biomarker with a VIP≥1.5 (**Figure 1C**), indicating its relevance to discriminate between true-LTECs and LTECs-LC. Moreover, LTECs-LC showed higher plasma sTNF-R1 values than true-LTECs (*p*=0.025; **Figure 1D**), difference that was maintained in a GLM adjusted by patient characteristics [aAMR=1.62 (95%CI=1.20; 2.19); *p*=0.001].

We also had access to a second plasma sample from 10 LTEC-LC patients, with a median time difference between the baseline and final sample of 3.9 (IQR=4.5) years. The type of HIV loss of control in this subgroup of LTECs-LC having a second plasma sample (n=10) was very similar to the one described in the first sample (20% virological, 60% immunological, and 20% virological and immunological). In the LTEC-LC group, we did not find significant differences in CD4+ T-cell counts between the two-time points (p>0.05). However, at the moment of this second LTECs-LC sample (n=10), these showed lower CD4^+^ T-cell counts than true-LTEC (n=36) (*p*=0.026; **Figure 1E**) similar HIV-1 viral load levels (**Figure 1F**), and again sTNF-R1 was the only plasma biomarker with VIP≥1.5 (**Figure 1G**). Moreover, sTNF-R1 was the only biomarker with significant differences between the study groups (*p*=0.005; **Figure 1H**), and the difference was maintained in an adjusted GLM [aAMR=1.93 (95%CI=1.41; 2.65); *p*<0.001]. The level of sTNF-R1 was significantly higher in the second plasma sample than the first plasma sample in LTECs-LC (Δx= 6.58 (95%=0.3; 12.88); *p*=0.040).

We also analyzed plasma sTNF-R1 values among the subgroup of LTECs with at least ten years of control of HIV-1 replication (20 true-LTECs *vs.* 16 LTECs-LC). At the moment of the first LTECs-LC samples, LTECs-LC had higher plasma sTNF-R1 values than true-LTECs (*p*=0.040). At the moment of the second LTECs-LC sample, LTECs-LC had higher plasma sTNF-R1 values than true-LTECs (*p*=0.016).

## Discussion

Factors associated with the loss of HIV-1 control remain unclear, and analyzing the evolution of HIV-LTECs may add relevant information. Our study showed that the plasma sTNF-R1 was related to the loss of natural HIV-1 control in our cohort of HIV-LTECs. Specifically, plasma sTNF-R1 was higher in LTEC-LC than true-LTEC in two different samples taken at other time points during follow-up in the group of LTECs-LC. Moreover, sTNF-R1 plasma levels increased over time in LTEC-LC patients, reinforcing the association of sTNF-R1 with the loss of natural HIV-1 control.

Among the general characteristics of the two study groups, the CD4^+^ T cell nadir count stands out, which was significantly different between the true-LTEC and LTEC-LC groups. The nadir CD4^+^ count was defined as the lowest CD4^+^ count during the follow-up of HIV-LTECs. Since the majority (75%) of LTEC-LC lost immunological control, it was not unexpected that the lowest CD4^+^ count during follow-up (the nadir CD4 T cell count) was lower in LTEC-LC than in true-LTEC.

Although HIV-LTECs may maintain sustained and robust immune responses for long periods (Navarrete-Munoz et al., 2020), several factors may promote the loss of spontaneous HIV-1 control (Pernas et al., 2018; Rosas-Umbert et al., 2019; Ruiz-Mateos et al., 2020). One of the critical factors in losing HIV-1 control is the activation and inflammation level (Pernas et al., 2018; Rosas-Umbert et al., 2019). In this regard, HIV-ECs that do not experience virological failure also present a persistent immune activation and high plasma levels of proinflammatory cytokines and chemokines, increasing the risk of non-AIDS-defining events (Pernas et al., 2018; Rosas-Umbert et al., 2019; Navarrete-Munoz et al., 2020; Ruiz-Mateos et al., 2020).

TNFα plays an essential role in the pathogenesis of HIV-1 (Kumar et al., 2016). Elevated amounts of TNFα have been detected in plasma and tissues at all stages of HIV-1 infection (von Sydow et al., 1991), even in patients on ART and undetectable viral load (Sereti et al., 2017). TNFα interacts with the cell transmembrane receptors TNF-R1 and TNF-R2, mainly to modulate immunity. The soluble form of TNF-R1 (sTNF-R1) is released by proteolytic cleavage or detachment of membrane receptors in exosomes (Levine, 2008), blocking the binding of TNFα to transmembrane TNF-R1 and inhibiting the TNFα activity (Van Zee et al., 1992). This TNFα/sTNF-R1 interaction regulates inflammation, and elevated sTNF-R1 values indicate increased inflammation and immune activation levels linked to viral replication and CD4^+^ T-cells depletion (Pasquereau et al., 2017). Besides, high sTNF-R1 levels are associated with loss of CD4^+^ T-cells in HIV-ECs with a sustained virological control (Gutierrez-Rivas et al., 2018) and HIV-1 individuals on ART (Richert et al., 2017), and with development of AIDS and non-AIDS events during suppressive ART (Kalayjian et al., 2010; Tenorio et al., 2014). To our knowledge, this study is the first suggesting an essential role for sTNF-R1 in the loss of natural HIV-1 control in the particular subpopulation of EC patients with long-term control of HIV-1. It is important to note that among the population of LTEC-LC patients, half of them lost immunological control (significant decrease of CD4^+^ T-cell counts over time) even though they maintained virological control, with the other half of patients losing virological control or both virological and immunological. These results suggest that sTNF-R1 may have a role in both types of loss of HIV-1 control. First, given the association of sTNF-R1 with HIV-1 plasma viremia (Rizzardi et al., 1996), the elevated levels of this marker in LTEC patients that will later lose virological control may reflect the existence of low levels of active viral replication below the limit of detection, but the lack of this data in our LTEC cohort precluded us from testing this hypothesis. On the other hand, the elevated sTNF-R1 levels in patients maintaining virological control but with a significant decline of CD4^+^ T-cell counts could reflect the existence of an increased state of immune activation or systemic inflammation (even in the face of undetectable viremia), a pathogenic mechanism that has been associated with CD4^+^ T-cells loss (Paiardini and Muller-Trutwin, 2013).

Lastly, our results may have potential clinical implications for managing HIV-LTECs, since it could guide when to initiate antiretroviral therapy in these HIV-1 patients to reduce residual viremia and immune activation (Ruiz-Mateos et al., 2020). However, sTNF-R1 is an inflammatory biomarker that is elevated in several disorders, including neurological disorders, end-stage renal and cardiovascular diseases, metabolic and rheumatic diseases, among others (Pegoretti et al., 2018; Cheng et al., 2020; Cudrici et al., 2020). These pathologies may be present in HIV-infected patients and could bias the ability of sTNF-R1 to discriminate HIV-LTECs. Therefore, the use of sTNF-R1 as a biomarker to differentiate between HIV-LTECs or decide to start antiretroviral therapy may not be suitable in those HIV patients suffering from these comorbidities.

Moreover, our study also has other limitations. First, the retrospective study design can introduce biases in the analysis and lack of uniformity. Second, this is a preliminary study with a limited sample size that may limit statistical power and increase the risk of false positives. However, it should be noted that these HIV-LTECs subjects are scarce. Third, the study includes many biomarkers and a small number of patients, which penalizes the finding of significant differences after adjusting for multiple comparisons. Fourth, our definition of HIV-LTECs could influence the generalization of our conclusions. However, despite these limitations, in our opinion, this study represents a relevant contribution in the field of HIV-ECs.

## Conclusions

In conclusion, high plasma values of sTNF-R1 appear to discriminate HIV-LTECs that lose the natural control of HIV-1, helping to define a specific phenotype that may be useful for the clinical management of these patients. Further analyses are needed to corroborate our findings and determine the long-term impact of increased sTNF-R1 in HIV-LTECs.

## Data Availability Statement

The raw data supporting the conclusions of this article will be made available by the authors, without undue reservation.

## Ethics Statement

The studies involving human participants were reviewed and approved by “Fundación Jiménez Díaz” Ethics Committee. The patients/participants provided their written informed consent to participate in this study.

## Author Contributions

Study conception and design: NR, JB, and SR. Acquisition of data and samples: all authors. Laboratory procedures: DS-C and MM-G. Analyses and interpretation of data: OB-K, SR, JB, and SR. Visualization and supervision: NR, JB, and SR. Drafting the article: DS-C and SR. Critical revision of the article: JB and NR. Funding acquisition: NR, JB, MM-F, and SR. All authors have read and approved the final manuscript.

## Funding

The study was also funded by the Spanish AIDS Research Network (RD16/0025/0013, RD16/0025/0019, and RD16CIII/0002/0002) and Centro de Investigación Biomédica en Red (CIBER) en Enfermedades Infecciosas (CB21/13/00044). NR is a ‘Miguel Servet’ researcher from the ISCIII (grant number CPII19/00025). DS-C is a ‘Sara Borrell’ researcher from the ISCIII (grant number CD20CIII/00001).

## Conflict of Interest

The authors declare that the research was conducted in the absence of any commercial or financial relationships that could be construed as a potential conflict of interest.

## Publisher’s Note

All claims expressed in this article are solely those of the authors and do not necessarily represent those of their affiliated organizations, or those of the publisher, the editors and the reviewers. Any product that may be evaluated in this article, or claim that may be made by its manufacturer, is not guaranteed or endorsed by the publisher.

## Appendix 1. Clinical Centers and research groups which contribute to ECRIS.

Clinical centers:

Hospital Universitario de Valme (Sevilla): Juan Antonio Pineda, Eva Recio Sánchez, Fernando Lozano de León, Juan Macías, José Carlos Palomares, Manuel Parra, Jesús Gómez-Mateos.

Hospital General Universitario Santa Lucía (Cartagena): Onofre Juan Martínez-Madrid, Francisco Vera, Lorena Martínez.

Hospital Clinic de Barcelona (Barcelona): José M. Miró, Christian Manzardo, Laura Zamora, Iñaki Pérez, Mª Teresa García, Carmen Ligero, José Luis Blanco, Felipe García-Alcaide, Esteban Martínez, Josep Mallolas, José M. Gatell.

Hospital General Universitario de Alicante (Alicante): Joaquín Portilla, Esperanza Merino, Sergio Reus, Vicente Boix, Livia Giner, Carmen Gadea, Irene Portilla, Maria Pampliega, Marcos Díez, Juan Carlos Rodríguez, Jose Sánchez-Payá.

Hospital Universitari de Bellvitge (Hospitalet de Llobregat): Daniel Podzamczer, Elena Ferrerm Arkaitz Imaz, Evan Van Den Eyncle, Silvana Di Yacovo, Maria Sumoy.

Hospital Universitario de Canarias (Santa Cruz de Tenerife): Juan Luis Gómez, Patricia Rodríguez, María Remedios Alemán, María del Mar Alonso, María Inmaculada Hernández, Felicitas Díaz-Flores, Dácil García, Ricardo Pelazas.

Hospital Carlos III (Madrid): Vicente Soriano, Pablo Labarga, Pablo Barreiro, Pablo Rivas, Francisco Blanco, Luz Martín Carbonero, Eugenia Vispo, Carmen Solera.

Hospital Universitario Central de Asturias (Oviedo): Victor Asensi, Eulalia Valle, José Antonio Cartón.

Hospital Doce de Octubre (Madrid): Rafael Rubio, Federico Pulido, Mariano Matarranz, Maria Lagarde, Guillermo Maestro, Rafael Rubio-Martín.

Hospital Universitario Donostia (San Sebastián): José Antonio Iribarren, Julio Arrizabalaga, María José Aramburu, Xabier Camino, Francisco Rodríguez-Arrondo, Miguel Ángel von Wichmann, Lidia Pascual Tomé, Miguel Ángel Goenaga, Mª Jesús Bustinduy, Harkaitz Azkune Galparsoro. Maialen Ibarguren, Mirian Aguado.

Hospital General Universitario de Elche (Elche): Félix Gutiérrez, Mar Masiá, Cristina López, Sergio Padilla, Andrés Navarro, Fernando Montolio, Catalina Robledano, Joan Gregori Colomé, Araceli Adsuar, Rafael Pascual, Federico Carlos, Maravillas Martinez.

Hospital Germans Trías i Pujol (Badalona): Roberto Muga, Jordi Tor, Arantza Sanvisens.

Hospital General Universitario Gregorio Marañón (Madrid): Juan Berenguer, Juan Carlos López Bernaldo de Quirós, Pilar Miralles, Isabel Gutiérrez, Margarita Ramírez, Belén Padilla, Paloma Gijón, Ana Carrero, Teresa Aldamiz-Echevarría, Francisco Tejerina, Francisco Jose Parras, Pascual Balsalobre, Cristina Diez.

Hospital Universitari de Tarragona Joan XXIII, IISPV, Universitat Rovira i Virgili (Tarragona): Francesc Vidal, Joaquín Peraire, Consuelo Viladés, Sergio Veloso, Montserrat Vargas, Miguel López-Dupla, Montserrat Olona, Alba Aguilar, Joan Josep Sirvent, Verónica Alba, Olga Calavia.

Hospital Universitario La Fe (Valencia): Marta Montero, José Lacruz, Marino Blanes, Eva Calabuig, Sandra Cuellar, José López, Miguel Salavert.

Hospital Universitario La Paz/IdiPaz (Madrid): Juan González, Ignacio Bernardino de la Serna, José Ramón Arribas, María Luisa Montes, Jose Mª Peña, Blanca Arribas, Juan Miguel Castro, Fco Javier Zamora, Ignacio Pérez, Miriam Estébanez, Silvia García, Marta Díaz, Natalia Stella Alcáriz, Jesús Mingorance, Dolores Montero, Alicia González, Maria Isabel de José.

Hospital de la Princesa (Madrid): Ignacio de los Santos, Jesús Sanz, Ana Salas, Cristina Sarriá, Ana Gómez.

Hospital San Pedro-CIBIR (Logroño): José Antonio Oteo, José Ramón Blanco, Valvanera Ibarra, Luis Metola, Mercedes Sanz, Laura Pérez-Martínez.

Complejo Hospitalario de Navarra (Pamplona): María Rivero, Marina Itziar Casado, Jorge Alberto Díaz, Javier Uriz, Jesús Repáraz, Carmen Irigoyen, María Jesús Arraiza.

Hospital Parc Taulí (Sabadell): Ferrán Segura, María José Amengual, Gemma Navarro, Montserrat Sala, Manuel Cervantes, Valentín Pineda, Victor Segura, Marta Navarro, Esperanza Antón, Mª Merce Nogueras.

Hospital Ramón y Cajal (Madrid): Santiago Moreno, José Luis Casado, Fernando Dronda, Ana Moreno, María Jesús Pérez Elías, Dolores López, Carolina Gutiérrez, Beatriz Hernández, Nadia Madrid, Angel Lamas, Paloma Martí, Alberto de Diaz, Sergio Serrano, Lucas Donat.

Hospital Reina Sofía (Murcia): Alfredo Cano, Enrique Bernal, Ángeles Muñoz.

Hospital San Cecilio (Granada): Federico García, José Hernández, Alejandro Peña, Leopoldo Muñoz, Jorge Parra, Marta Alvarez, Natalia Chueca, Vicente Guillot, David Vinuesa, Jose Angel Fernández.

Centro Sanitario Sandoval (Madrid): Jorge Del Romero, Carmen Rodríguez, Teresa Puerta, Juan Carlos Carrió, Cristina González, Mar Vera, Juan Ballesteros.

Hospital Son Espases (Palma de Mallorca): Melchor Riera, María Peñaranda, María Leyes, M° Angels Ribas, Antoni A Campins, Carmen Vidal, Leire Gil, Francisco Fanjul, Carmen Matinescu.

Hospital Universitario Virgen del Rocío (Sevilla): Manuel Leal, Pompeyo Viciana, Luis Fernando López-Cortés, Nuria Espinosa.

Research groups:

IIS-Fundación Jimenez Díaz, UAM. Jose Miguel Benito, Norma Rallón, Clara Restrepo, Noelia Rodriguez, Marcial García, Alfonso Cabello, Miguel Gorgolas.

Infección viral e Inmunidad. ISCIII. Salvador Resino, Veronica Briz, Maria Angeles Jimenez, Maria Sonia Vazquez, Amanda Fernandez, Pilar García.

Hospital Gregorio Marañón. Maria Angeles Muñoz, Javier Sanchez Rodriguez, Jose Luis Jimenez, Daniel Sepúlveda, Isabel García Merino, Irene Consuegra.

Hospital Clinic. Agathe León, Mireia Arnedo, Montse Plana, Nuria Climent, Felipe García.

Hospital Joan XXIII. Paco Vidal, Esther Rodriguez-Gallego, Consuelo Viladés, Joaquin Peraire

Centro Sandoval. Jorge Del Romero, Carmen Rodríguez, Mar Vera.

Fundacion IRSI CAIXA José Esté, Esther Ballana, Miguel Angel Martinez, S Franco,María Nevot.

Hospital Ramón y Cajal. Alejandro Vallejo, Beatriz Sara Sastre, Santiago Moreno.

Virologia Molecular ISCIII. Maria Pernas, Concepción Casado, Cecilio López Galíndez

Inmunopatología del SIDA. ISCIII. Laura Capa, Mayte Perez-Olmeda, Pepe Alcami

Mutacion y evolución de virus. Univ Valencia. Rafael Sanjuán, José Manuel Cuevas

Hospital 12 de Octubre. Rafael Delgado, Olalla Sierra

Universidad de la Laguna. Agustín Valenzuela-Fernández.

Hospital Virgen del Rocio: Ezequiel Ruiz-Mateos, Beatriz Dominguez-Molina, Laura Tarancón-Diez, Mohamed Rafii-El-Idrissi Benhnia, Maria José Polaino, Miguel Genebat, Pompeyo Viciana, Manuel Leal.
