# Supplementary material for: High Plasma sTNF-R1 Level Is Related to Loss of Natural HIV Control in Long-Term Elite Controllers
Source: Front Cell Infect Microbiol. 2022 Mar 17;12:858872. doi: 10.3389/fcimb.2022.858872 (PMC8968196; doi:10.3389/fcimb.2022.858872)
Supplement: Supplementary file 1 [file DataSheet_1.docx]

**Appendix 1.** Clinical Centers and research groups which contribute to ECRIS.

Clinical centers:

Hospital Universitario de Valme (Sevilla): Juan Antonio Pineda, Eva Recio Sánchez, Fernando Lozano de León, Juan Macías, José Carlos Palomares, Manuel Parra, Jesús Gómez-Mateos.

Hospital General Universitario Santa Lucía (Cartagena): Onofre Juan Martínez-Madrid, Francisco Vera, Lorena Martínez.

Hospital Clinic de Barcelona (Barcelona): José M. Miró, Christian Manzardo, Laura Zamora, Iñaki Pérez, Mª Teresa García, Carmen Ligero, José Luis Blanco, Felipe García-Alcaide, Esteban Martínez, Josep Mallolas, José M. Gatell.

Hospital General Universitario de Alicante (Alicante): Joaquín Portilla, Esperanza Merino, Sergio Reus, Vicente Boix, Livia Giner, Carmen Gadea, Irene Portilla, Maria Pampliega, Marcos Díez, Juan Carlos Rodríguez, Jose Sánchez-Payá.

Hospital Universitari de Bellvitge (Hospitalet de Llobregat): Daniel Podzamczer, Elena Ferrerm Arkaitz Imaz, Evan Van Den Eyncle, Silvana Di Yacovo, Maria Sumoy.

Hospital Universitario de Canarias (Santa Cruz de Tenerife): Juan Luis Gómez, Patricia Rodríguez, María Remedios Alemán, María del Mar Alonso, María Inmaculada Hernández, Felicitas Díaz-Flores, Dácil García, Ricardo Pelazas.

Hospital Carlos III (Madrid): Vicente Soriano, Pablo Labarga, Pablo Barreiro, Pablo Rivas, Francisco Blanco, Luz Martín Carbonero, Eugenia Vispo, Carmen Solera.

Hospital Universitario Central de Asturias (Oviedo): Victor Asensi, Eulalia Valle, José Antonio Cartón.

Hospital Doce de Octubre (Madrid): Rafael Rubio, Federico Pulido, Mariano Matarranz, Maria Lagarde, Guillermo Maestro, Rafael Rubio-Martín.

Hospital Universitario Donostia (San Sebastián): José Antonio Iribarren, Julio Arrizabalaga, María José Aramburu, Xabier Camino, Francisco Rodríguez-Arrondo, Miguel Ángel von Wichmann, Lidia Pascual Tomé, Miguel Ángel Goenaga, Mª Jesús Bustinduy, Harkaitz Azkune Galparsoro. Maialen Ibarguren, Mirian Aguado.

Hospital General Universitario de Elche (Elche): Félix Gutiérrez, Mar Masiá, Cristina López, Sergio Padilla, Andrés Navarro, Fernando Montolio, Catalina Robledano, Joan Gregori Colomé, Araceli Adsuar, Rafael Pascual, Federico Carlos, Maravillas Martinez.

Hospital Germans Trías i Pujol (Badalona): Roberto Muga, Jordi Tor, Arantza Sanvisens.

Hospital General Universitario Gregorio Marañón (Madrid): Juan Berenguer, Juan Carlos López Bernaldo de Quirós, Pilar Miralles, Isabel Gutiérrez, Margarita Ramírez, Belén Padilla, Paloma Gijón, Ana Carrero, Teresa Aldamiz-Echevarría, Francisco Tejerina, Francisco Jose Parras, Pascual Balsalobre, Cristina Diez.

Hospital Universitari de Tarragona Joan XXIII, IISPV, Universitat Rovira i Virgili (Tarragona): Francesc Vidal, Joaquín Peraire, Consuelo Viladés, Sergio Veloso, Montserrat Vargas, Miguel López-Dupla, Montserrat Olona, Alba Aguilar, Joan Josep Sirvent, Verónica Alba, Olga Calavia.

Hospital Universitario La Fe (Valencia): Marta Montero, José Lacruz, Marino Blanes, Eva Calabuig, Sandra Cuellar, José López, Miguel Salavert.

Hospital Universitario La Paz/IdiPaz (Madrid): Juan González, Ignacio Bernardino de la Serna, José Ramón Arribas, María Luisa Montes, Jose Mª Peña,  Blanca Arribas, Juan Miguel Castro, Fco Javier Zamora, Ignacio Pérez, Miriam Estébanez, Silvia García, Marta Díaz, Natalia Stella Alcáriz, Jesús Mingorance, Dolores Montero, Alicia González, Maria Isabel de José.

Hospital de la Princesa (Madrid): Ignacio de los Santos, Jesús Sanz, Ana Salas, Cristina Sarriá, Ana Gómez.

Hospital San Pedro-CIBIR (Logroño): José Antonio Oteo, José Ramón Blanco, Valvanera Ibarra, Luis Metola, Mercedes Sanz, Laura Pérez-Martínez.

Complejo Hospitalario de Navarra (Pamplona): María Rivero, Marina Itziar Casado, Jorge Alberto Díaz, Javier Uriz, Jesús Repáraz, Carmen Irigoyen, María Jesús Arraiza.

Hospital Parc Taulí (Sabadell): Ferrán Segura, María José Amengual, Gemma Navarro, Montserrat Sala, Manuel Cervantes, Valentín Pineda, Victor Segura, Marta Navarro, Esperanza Antón, Mª Merce Nogueras.

Hospital Ramón y Cajal (Madrid): Santiago Moreno, José Luis Casado, Fernando Dronda, Ana Moreno, María Jesús Pérez Elías, Dolores López, Carolina Gutiérrez, Beatriz Hernández, Nadia Madrid, Angel Lamas, Paloma Martí, Alberto de Diaz, Sergio Serrano, Lucas Donat.

Hospital Reina Sofía (Murcia): Alfredo Cano, Enrique Bernal, Ángeles Muñoz.

Hospital San Cecilio (Granada): Federico García, José Hernández, Alejandro Peña, Leopoldo Muñoz, Jorge Parra, Marta Alvarez, Natalia Chueca, Vicente Guillot, David Vinuesa, Jose Angel Fernández.

Centro Sanitario Sandoval (Madrid): Jorge Del Romero, Carmen Rodríguez, Teresa Puerta, Juan Carlos Carrió, Cristina González, Mar Vera, Juan Ballesteros.

Hospital Son Espases (Palma de Mallorca): Melchor Riera, María Peñaranda, María Leyes, Mº Angels Ribas, Antoni A Campins, Carmen Vidal, Leire Gil, Francisco Fanjul, Carmen Matinescu.

Hospital Universitario Virgen del Rocío (Sevilla): Manuel Leal, Pompeyo Viciana, Luis Fernando López-Cortés, Nuria Espinosa.

Research groups:

IIS-Fundación Jimenez Díaz, UAM. Jose Miguel Benito, Norma Rallón, Clara Restrepo, Noelia Rodriguez, Marcial García, Alfonso Cabello, Miguel Gorgolas.

Infección viral e Inmunidad. ISCIII. Salvador Resino, Veronica Briz, Maria Angeles Jimenez, Maria Sonia Vazquez, Amanda Fernandez, Pilar García.

Hospital Gregorio Marañón. Maria Angeles Muñoz, Javier Sanchez Rodriguez, Jose Luis Jimenez, Daniel Sepúlveda, Isabel García Merino, Irene Consuegra.

Hospital Clinic. Agathe León, Mireia Arnedo, Montse Plana, Nuria Climent, Felipe García.

Hospital Joan XXIII. Paco Vidal, Esther Rodriguez-Gallego, Consuelo Viladés, Joaquin Peraire

Centro Sandoval. Jorge Del Romero, Carmen Rodríguez, Mar Vera.

Fundacion IRSI CAIXA José Esté, Esther Ballana, Miguel Angel Martinez, S Franco,María Nevot.

Hospital Ramón y Cajal. Alejandro Vallejo, Beatriz Sara Sastre, Santiago Moreno.

Virologia Molecular ISCIII. Maria Pernas, Concepción Casado, Cecilio López Galíndez

Inmunopatología del SIDA. ISCIII. Laura Capa, Mayte Perez-Olmeda, Pepe Alcami

Mutacion y evolución de virus. Univ Valencia. Rafael Sanjuán, José Manuel Cuevas

Hospital 12 de Octubre. Rafael Delgado, Olalla Sierra

Universidad de la Laguna. Agustín Valenzuela-Fernández.

Hospital Virgen del Rocio: Ezequiel Ruiz-Mateos, Beatriz Dominguez-Molina, Laura Tarancón-Diez, Mohamed Rafii-El-Idrissi Benhnia, Maria José Polaino, Miguel [Genebat](http://www.ncbi.nlm.nih.gov/pubmed/?term=Genebat%20M), Pompeyo [V](http://www.ncbi.nlm.nih.gov/pubmed/?term=Viciana%20P)iciana, Manuel Leal.
